# The auxin response factor gene family in banana: genome-wide identification and expression analyses during development, ripening, and abiotic stress

**DOI:** 10.3389/fpls.2015.00742

**Published:** 2015-09-15

**Authors:** Wei Hu, Jiao Zuo, Xiaowan Hou, Yan Yan, Yunxie Wei, Juhua Liu, Meiying Li, Biyu Xu, Zhiqiang Jin

**Affiliations:** ^1^Key Laboratory of Biology and Genetic Resources of Tropical Crops, Institute of Tropical Bioscience and Biotechnology, Chinese Academy of Tropical Agricultural SciencesHaikou, China; ^2^Key Laboratory of Genetic Improvement of Bananas, Haikou Experimental Station, Chinese Academy of Tropical Agricultural SciencesHaikou, China

**Keywords:** auxin response factors (ARF) transcription factor, abiotic stresses, banana, development, expression analysis, ripening

## Abstract

Auxin signaling regulates various auxin-responsive genes via two types of transcriptional regulators, Auxin Response Factors (ARF) and Aux/IAA. ARF transcription factors act as critical components of auxin signaling that play important roles in modulating various biological processes. However, limited information about this gene family in fruit crops is currently available. Herein, 47 *ARF* genes were identified in banana based on its genome sequence. Phylogenetic analysis of the ARFs from banana, rice, and *Arabidopsis* suggested that the ARFs could be divided into four subgroups, among which most ARFs from the banana showed a closer relationship with those from rice than those from *Arabidopsis*. Conserved motif analysis showed that all identified MaARFs had typical DNA-binding and ARF domains, but 12 members lacked the dimerization domain. Gene structure analysis showed that the number of exons in *MaARF* genes ranged from 5 to 21, suggesting large variation amongst banana *ARF* genes. The comprehensive expression profiles of *MaARF* genes yielded useful information about their involvement in diverse tissues, different stages of fruit development and ripening, and responses to abiotic stresses in different varieties. Interaction networks and co-expression assays indicated the strong transcriptional response of banana ARFs and ARF-mediated networks in early fruit development for different varieties. Our systematic analysis of MaARFs revealed robust tissue-specific, development-dependent, and abiotic stress-responsive candidate *MaARF* genes for further functional assays *in planta*. These findings could lead to potential applications in the genetic improvement of banana cultivars, and yield new insights into the complexity of the control of *MaARF* gene expression at the transcriptional level. Finally, they support the hypothesis that ARFs are a crucial component of the auxin signaling pathway, which regulates a wide range of physiological processes.

## Introduction

The phytohormone auxin plays crucial roles in various aspects of plant growth and development, such as lateral root initiation, apical dominance, shoot elongation, embryo patterning, and vascular differentiation (Davies, 1995). A growing body of evidence indicates that auxin, either alone or together with other hormones, is involved in plant responses to environmental stimuli, including drought, cold, and salt (Park et al., 2007; Zhang et al., 2009; Zahir et al., 2010; Du et al., 2012; Lee et al., 2012). Accumulated evidences have established the basic model of auxin pathway from perception to gene expression, showing that ubiquitination of indole-3-acetic acid-inducible (Aux/IAA) proteins by the TIR1/AFB subunit of the SCF^TIR1/AFB^ ubiquitin ligase results in their degradation by the 26S proteasome, thus releasing the Aux/IAA-mediated inhibition of ARFs to modulate the expression of their target genes, such as SMALL AUXIN UP RNA (*SAUR*), Gretchen Hagen 3 (*GH3*), and indole-3-acetic acid-inducible gene (*Aux/IAA*) (Hayashi, 2012). Auxin response factors (ARFs), important transcription factors in the auxin signaling pathway, regulate the transcription of auxin-responsive genes by directly binding to the auxin response element (AuxRE, TGTCTC) of their promoters (Hagen and Guilfoyle, 2002). ARF proteins contain three unique domains: a conserved N-terminal DNA-binding domain (DBD), a variable middle transcriptional regulatory region (MR), and a C-terminal dimerization domain (CTD) (Pérez-Rodríguez, et al., 2010; Zhang et al., 2011). The DBD domain can specifically target the AuxRE element in the promoter of auxin-responsive genes (Hagen and Guilfoyle, 2002; Ha et al., 2013). The MR domain acts to induce transcriptional activation or repression based on the composition of its amino acid residues (Ulmasov et al., 1997, 1999). The CTD domain, similar to domains III and IV of Aux/IAA proteins, is involved in protein-protein interactions, mediating the homodimerization of ARFs and the heterodimerization of ARF and Aux/IAA proteins (Tiwari et al., 2003; Guilfoyle and Hagen, 2007).

Since the first *ARF* gene (*AtARF1*) was cloned from *Arabidopsis thaliana*, genome-wide analyses have identified 23 ARFs from *Arabidopsis* (Okushima et al., 2005), 25 ARFs from rice (Wang et al., 2007), 39 ARFs from *Populus trichocarpa* (Kalluri et al., 2007), 24 ARFs from sorghum (Paterson et al., 2009), 31 ARFs from maize (Xing et al., 2011), 22 ARFs from tomato (Kumar et al., 2011; Zouine et al., 2014), 31 ARFs from *Brassica rapa* (Mun et al., 2012), 51 ARFs from soybean (Ha et al., 2013), 17 ARFs from *Eucalyptus grandis* (Yu et al., 2014), 24 ARFs from *Medicago truncatula* (Shen et al., 2015), and 19 ARFs from sweet orange (Li et al., 2015). Furthermore, biochemical and genetic analyses have established a crucial function for *ARF* genes in plant growth and development. For example, T-DNA insertion and EMS mutation analyses revealed that *AtARF2* could regulate floral organ abscission, leaf senescence, and seed size and weight (Ellis et al., 2005; Schruff et al., 2005; Lim et al., 2010). An *arf19arf7* double-mutant exhibited strong auxin resistance, while *arf19* also exhibited an ethylene-insensitive phenotype in *Arabidopsis* roots, suggesting that the integration of hormonal signals by *ARFs* can regulate plant growth and development (Li et al., 2006). The mutation of *AtARF5* in *Arabidopsis* caused the vascular development and early embryo formation (Hardtke and Berleth, 1998). The mutation in *AtARF8* resulted in the uncoupling of fruit development from pollination and fertilization, and yielded seedless fruit (Goetz et al., 2006). Other findings have also shown extensive responses of *ARF* genes to abiotic stresses. Genome-wide identification and expression analyses of the *ARF* gene family suggest that the expression of many *ARF* genes are altered in various species in responsive to abiotic stresses, such drought, salt, or cold (Jain and Khurana, 2009; Wang et al., 2010; Ha et al., 2013; Sekhwal et al., 2015). Recently, tasiRNA-ARF was reported to be involved in maintaining the normal morphogenesis of flowers under stress conditions by fine-tuning changes in the expression of floral development- and auxin response-related genes in *Arabidopsis* (Matsui et al., 2014). Together, these studies have shown that the *ARF* gene family is involved in regulating plant growth and development, and in plant responses to multiple signaling transduction pathways.

The banana (*Musa acuminata* L.) is a large annual monocotyledonous herbaceous plant found in tropical and subtropical climates. Globally, it is one of the most popular fresh fruits. The banana is also the second largest fruit crop and one of the top five food commodities worldwide (Sreedharan et al., 2013). Compared to other important food crops, studies of the banana proceeded slowly because the banana is only a staple food for the largely impoverished continent of Africa (Sreedharan et al., 2013). Banana plants are frequently destroyed by various biotic and abiotic stresses during their growth and development. Investigations of metabolic pathways and signal transduction pathways based on complete genome sequences are necessary to elucidate both cellular and organismal processes (Hirayama and Shinozaki, 2010). However, few studies on identification and characterization of banana genes based on banana genome sequencing data have been reported (Asif et al., 2014; Backiyarani et al., 2015). Considering the importance of the *ARF* gene family in both plant growth and development as well as responses to diverse environmental stresses, we decided to conduct a genome-wide analysis of the banana genome. Herein, we identified 47 MaARFs and analyzed their phylogenetic relationship, gene structure, protein motifs, interaction networks. Further, we also characterized the expression patterns of *MaARF* genes in diverse tissues, different stages of fruit development and ripening, and the response to abiotic stresses in different banana varieties. Our identification and comprehensive characterization of the ARF family in banana establish an important foundation for future studies aimed at crop improvement and understanding abiotic stresses responses medicated by ARFs in plants.

## Materials and methods

### Plant materials and treatments

BaXi Jiao (*Musa acuminate* L. AAA group cv. Cavendish, BX), which is a high quality fruit because of its high yield, long fingers, and long-term storage, is widely cultivated and accounts for large amount of the cultivated area in China. Fen Jiao (*Musa* ABB Pisang Awak, FJ), which has the characteristics of good flavor and tolerance to abiotic stresses, has been widely cultivated in the Hainan province of China. Young banana seedlings of BX and FJ with uniform growth during the five-leaf stage were obtained from the banana tissue culture center (Danzhou, Institute of Bananas and Plantains, Chinese Academy of Tropical Agricultural Sciences). Banana seedlings were grown in soil under greenhouse conditions (28°C; 200 μmol m^−2^ s^−1^ light intensity; 16 h light/8 h dark cycle; 70% relative humidity) or planted at a banana-planting base (Wenchang, Institute of Tropical Bioscience and Biotechnology, Chinese Academy of Tropical Agricultural Sciences).

### Identification and phylogenetic analyses of the MaARF gene family in banana

The whole protein sequence for the banana (*Musa acuminata*) was downloaded from the banana genome database (http://banana-genome.cirad.fr/) (D'Hont et al., 2012). The ARF amino acid sequences from rice and *Arabidopsis* were acquired from the RGAP (http://rice.plantbiology.msu.edu/) and UniPort (http://www.uniprot.org/) databases, respectively. A Hidden Markov Model (HMMER: http://hmmer.janelia.org/) built from known ARFs was used to query the predicted ARF proteins based on the banana genome sequence (Finn et al., 2011). The possible ARFs in banana database were further characterized by BLAST analysis using all ARFs from rice and *Arabidopsis* as queries. All identified ARFs were further validated by a conserved domain search using the CDD (http://www.ncbi.nlm.nih.gov/cdd/) and PFAM (http://pfam.sanger.ac.uk/) databases. Phylogenetic analysis was carried out according to the neighbor-joining (NJ) method using the ClustalX 2.0 and MEGA 5.0 software packages (Tamura et al., 2011).

### Protein properties and sequence analyses

The relative molecular mass and isoelectric points of the identified MaARFs were predicted using the ExPASy proteomics server database (http://expasy.org/). Motifs of MaARF proteins were analyzed using MEME software (http://meme.nbcr.net/meme/cgi-bin/meme.cgi). The number of motifs was 15 and the optimum motif width was between 8 and 50. The predicted motifs of MaARFs were further annotated using an InterProScan database search (http://www.ebi.ac.uk/Tools/pfa/iprscan/). The structural features of the MaARF genes were identified using GSDS software (http://gsds.cbi.pku.edu.cn/) based on the genome and coding sequences of MaARFs. Those interactions between ARFs and their interacting proteins that were supported by experimental evidence were determined using STRING (http://string-db.org/).

### Transcriptomic analysis

Banana leaves at the five-leaf stage, roots, and fruits 80 days after flowering (DAF) in BX and FJ varieties were sampled to detect the expression profiles of MaARF genes in various tissues. In the fruit development process for the banana, fruits of 0, 20, and 80 DAF represent fruits at the coming out a bud, cutting flower, and full-grown/harvest stages, respectively. Thus, fruits of 0, 20, and 80 DAF in BX and FJ varieties were sampled to assess the expression patterns of MaARF genes during fruit development. In the post-harvest ripening process for banana, the degree of ripening can be divided into the following seven stages according to Pua et al. (2003): full green (FG), trace yellow (TY), more green than yellow (MG), more yellow than green (MY), green tip (GT), full yellow (FY), and yellow flecked with brown spots (YB). Fruits of 8 and 14 days postharvest (DPH) in BX reached MG and FY degrees, respectively, whereas those of 3 and 6 DPH in FJ reached MG and FY degrees, respectively. Therefore, fruits at 8 and 14 DPH in BX and at 3 and 6 DPH in FJ were sampled to examine the expression patterns of MaARF genes in the post-harvest ripening process. For osmotic and salt treatments, banana seedlings at the five-leaf stage grown in soil were irrigated with 200 mM mannitol or 300 mM NaCl for 7 days, respectively. For cold treatment, banana seedlings were cultured in a growth chamber with a temperature that was maintained at 4°C for 22 h. The global expression patterns of banana genes were determined using RNA-seq. Total RNA was isolated from the following sources: different tissues of leaves, roots, and fruits; different developmental and ripening stages of fruits; and leaves of banana seedlings under normal or abiotic stresses treatments in BX and FJ varieties using a plant RNA extraction kit (TIANGEN, China). A total of 3 μg total RNA from each sample was converted into cDNA using a RevertAid First-Strand cDNA Synthesis Kit (Fermentas). The cDNA libraries were constructed according to the protocols supplied by Illumina, and were subsequently subjected to sequencing with an Illumina GAII following the Illumina RNA-seq protocol. Each sample contained two biological replicates. The genome sequence of DH-Pahang (*Musa acuminate*, A-genome, 2*n* = 22) was used as a reference genome for analyses of transcriptomic data (D'Hont et al., 2012).

## Results

### Identification and phylogenetic analysis of banana ARFs

To identify all ARF family members in banana genome, both BLAST and Hidden Markov Model searches were performed to search the banana genome database using *Arabidopsis* and rice ARF sequences as queries. A total of 47 non-redundant *MaARF* genes were identified in the banana genome, and these classifications were supported by conserved domain and multiple sequence alignment analyses. The 47 predicted full-length banana ARF proteins ranged from 448 (MaARF26) to 1067 (MaARF22) amino acid residues and the relative molecular mass varied from 49.209 (MaARF26) to 118.81 (MaARF22) kDa, with PIs in the range of 5.25–9.4, suggesting that they might act in different subcellular environments (Table S1).

**Figure 1:**
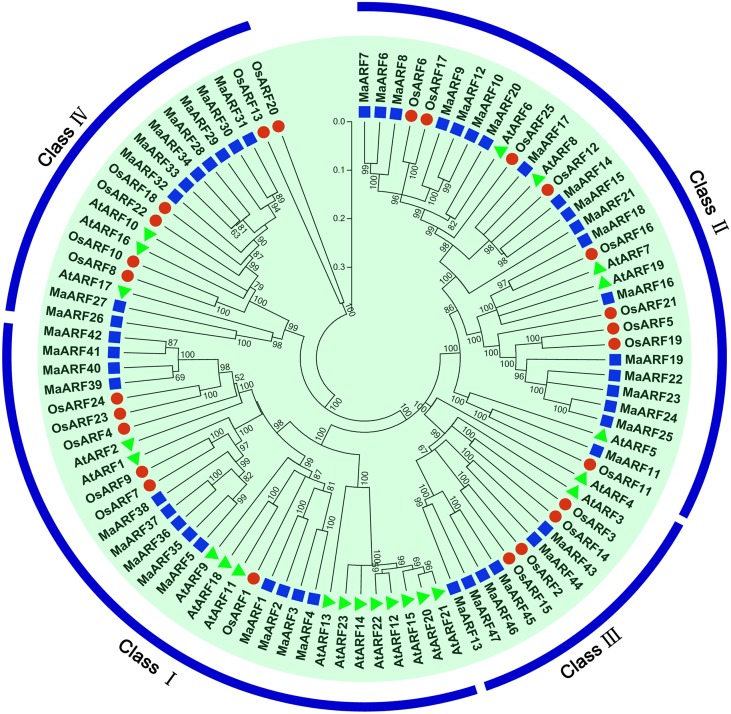
**Phylogenetic analysis of ARFs from Arabidopsis, rice, and banana using Neighbor-joining method**. The phylogenetic tree was produced by using Clustal X 2.0 and MEGA 5.0 softwares with the pair-wise deletion option. Tree reliability was assessed using 1000 bootstrap replicates. The numbers indicated for each clade represent bootstrap support values given as percentages. Four subgroups were shown as I, II, III, and IV. Solid square, 47 ARF proteins from banana; Solid round, 25 ARF proteins from rice; Solid triangle, 23 ARF proteins from Arabidopsis.

To characterize the evolutionary relationship between banana ARF proteins and other ARFs, a neighbor-joining tree was generated based on alignments of ARF family proteins from banana, rice, and *Arabidopsis* (Figure 1). The results indicated that the 47 identified MaARFs could be assigned to the following four separate clusters (I, II, III, and IV), together with ARFs from *Arabidopsis* and rice: Cluster I included MaARF-1, -2, -3, -4, -5, -35, -36, -37, -38, -39, -40, -41, and -42; Cluster II included MaARF-6, -7, -8, -9, -10, -11, -12, -14, -15, -16, -17, -18, -19, -20, -21, -22, -23, -24, and -25; Cluster III included MaARF-13, -43, -44, -45, -46, and -47; and Cluster IV included MaARF-26, -27, -28, -29, -30, -31, -32, -33, and -34.

### Gene structure and conserved motifs of banana ARFs

**Figure 2:**
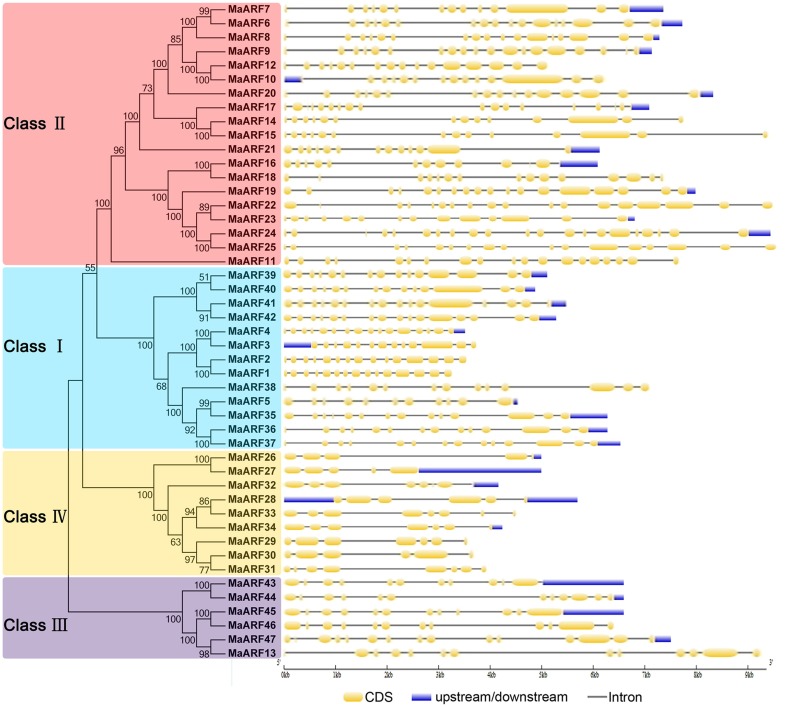
**The phylogenetic relationship and exon-intron structure analyses of banana ARFs**. The Neighbor-joining tree was created using ClustalX 2.0 and MEGA5 with amino acid sequences of MaARFs. Four clusters labeled as I, II, III, and IV are indicated with different color backgrounds. Exon-intron structure analyses were performed by GSDS database. Lengths of exons and introns of each MaARF gene were exhibited proportionally.

To better understand the structural evolution of the *MaARF* genes, the exon–intron features of banana ARFs were analyzed based on an evolutionary analysis of the MaARFs. Our evolutionary analysis confirmed the classification of the 47 banana ARFs into four groups (Figure 2), which was in accord with our clustering analysis data in Figure 1. Gene structure analysis showed that the *MaARF* genes contained 11–16 exons in cluster I, 11–21 exons in cluster II, 11–13 exons in cluster III, and 5–8 exons in cluster IV. This finding suggested that the number of exons for the *MaARF* genes was greater in clusters I–III than in cluster IV.

**Figure 3:**
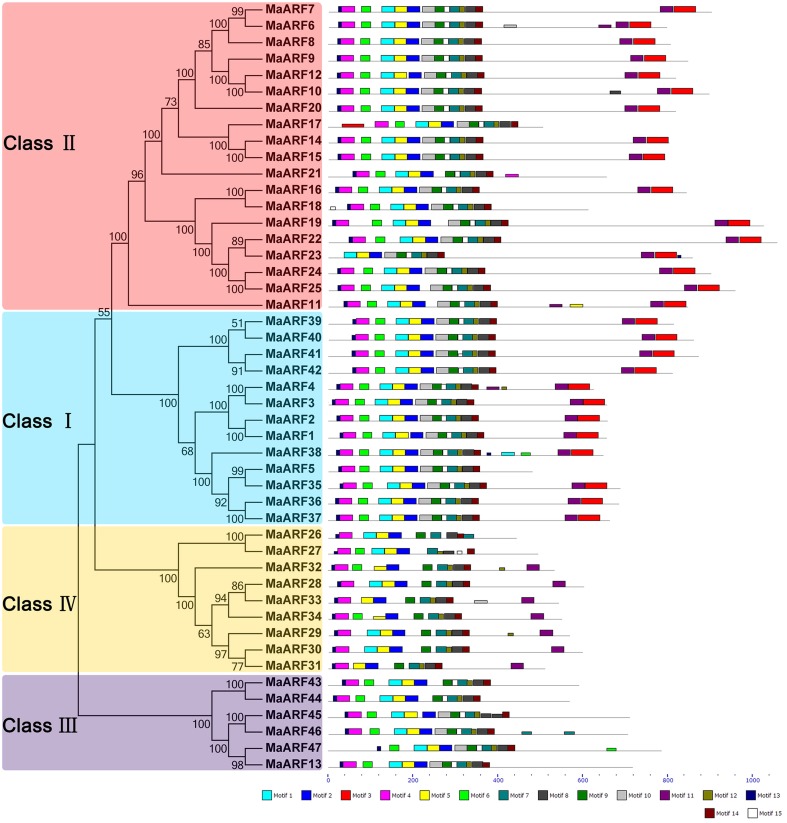
**The phylogenetic relationship and motif analyses of banana ARFs**. The Neighbor-joining tree was created using ClustalX 2.0 and MEGA5 with amino acid sequences of MaARFs. Four clusters labeled as I, II, III, and IV are indicated with different color backgrounds. All motifs were identified by MEME database with the complete amino acid sequences of MaARFs. Lengths of motifs for each MaARF protein were exhibited proportionally.

To explore the structural diversity and functional prediction of banana ARFs, a motif analysis was performed based on the phylogenetic relationship (Figure 3, Table S2). A total of 15 conserved motifs in the MaARFs were identified using MEME software and annotated with the InterPro database. Motifs 1 and 5 were annotated as DNA-binding pseudobarrel domain; motif 2 was annotated as DNA-binding pseudobarrel and B3 DNA binding domains; motifs 3 and 11 were annotated as AUX/IAA protein and Aux/IAA-ARF-dimerization; and motifs 6 and 8 were annotated as auxin response factors. According to the motif analysis and annotation, all identified MaARFs contained a typical ARF-type structure with a conserved DBD that consisted of a plant-specific B3 DNA binding motif (motif 2) and ARF motifs (motifs 6 and 8). For the CTD domain, most MaARFs (35/41, 85.4%) in clusters I, II, and IV have a CTD domain with motif 3 and/or 11, which was annotated as AUX/IAA protein and Aux/IAA-ARF-dimerization, whereas all MaARFs in cluster III lacked a CTD domain. Therefore, 25.5% (12/47) of MaARFs lacked a CTD domain. Moreover, most MaARF members in clusters I and II had both motifs 3 and 11, whereas most MaARF members in cluster IV only contained motif 11. Additionally, some closely related MaARFs had a similar motif structure, such as MaARF-14 and -15, MaARF-24 and -25, MaARF-39 and -40, and MaARF-41 and -42. Overall, most conserved motifs existed in the same subgroup, suggesting that the classification of MaARFs correlated with their amino acid sequences. Thus, the phylogenetic relationships and classification of banana ARFs presented above were further supported by this motif analysis.

### Expression patterns of *MaARF* genes in different tissues of two banana varieties

**Figure 4:**
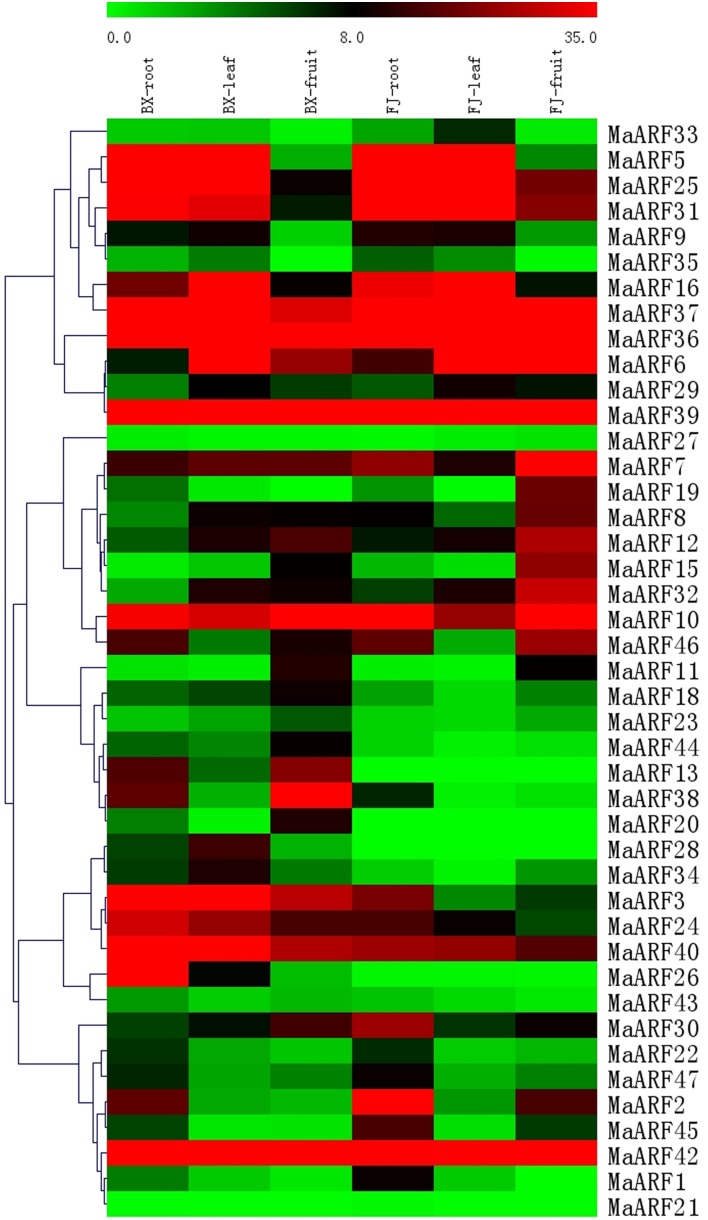
**Expression analysis of MaARF genes in roots, leaves, and fruits of two banana varieties**. Banana leaves and roots at the five-leaf stage and fruits at 80 DAF in the BX and FJ varieties were sampled to detect the expression profiles of MaARF genes in various tissues. The heat map was created based on the transcriptomic data of ARFs from two independent experiments. The scale represents the signal intensity values.

A large body of evidence supports the importance of *ARF* genes in plant growth and development (Ellis et al., 2005; Schruff et al., 2005; Guilfoyle and Hagen, 2007; Lim et al., 2010). Thus, we examined changes in *MaARF* gene expression in different tissues at the transcriptional level to seek insights into the roles of *MaARFs* in banana growth and development. Tissues of roots, leaves, and fruits in BX and FJ were sampled to assess the expression patterns of the *MaARF* genes. By transcriptomic analyses, we obtained transcript data for 43 *MaARF* genes in different tissues (Figure 4, Table S3).

For BX, all 43 genes (100%) were expressed in different tissues, among which 18 (41.9%), 18 (41.9%), and 17 (39.5%) genes showed high expression levels (value > 10) in root, leaf, and fruit tissues, respectively. Additionally, there were 9 genes (MaARF-3, -7, -10, -24, -36, -37, -39, -40, and -42) that showed high expression levels (value > 10) in all tissues examined.

For FJ, 41 (95.4%), 39 (90.7%), and 40 (93.0%) genes were expressed in root, leaf, and fruit tissues, respectively, among which 19 (44.2%), 15 (34.9%), and 17 (39.5%) genes showed high expression levels (value > 10). There were 10 genes (MaARF-6, -7, -10, -25, -31, -36, -37, -39, -40, and -42) that had high expression levels (value > 10) in all organs tested. *MaARF-28* and *-13* did not show expression in root, leaf, or fruit tissues; *MaARF-20* did not show expression in leaf and root tissues; and *MaARF-21* did not show expression in leaves.

To compare the expression profiles of *MaARFs* in different tissues of BX and FJ, 43 genes (100%) showed expression in all BX tissues, whereas 39 genes (90.7%) showed expression in all FJ tissues. The absence of detection might reflect distinct temporal or spatial expression patterns for these genes. Generally, *MaARFs* showed similar expression profiles in BX and FJ roots and leaves, suggesting that *MaARF* genes played similar roles in these two tissues of BX and FJ. However, some genes showed differential expression patterns in BX and FJ fruits, including 7 genes (MaARF-2, -8, -15, -19, -25, -31, and -32) that showed high expression levels (value > 15) in FJ fruits, whereas low expression levels (value < 10) were detected in BX fruits. There were 5 genes (MaARF-3, -13, -24, -30, and -38) that showed high expression levels (value > 15) in BX fruits, whereas low expression levels (value < 10) were detected in FJ fruits. These findings suggested differential roles for these genes in fruit development in different banana varieties. Additionally, 7 genes (*MaARF-7, -10, -36, -37, -39, -40*, and *-42*) showed high expression levels (value > 10) in all BX and FJ tissues, indicating key roles for these genes in tissue development. Together, these tissue expression profiles of *ARF* genes in different varieties may provide insights for future studies of tissue development and function.

### Expression profiles of *MaARF* genes in different stages of fruit development and ripening from two banana varieties

**Figure 5:**
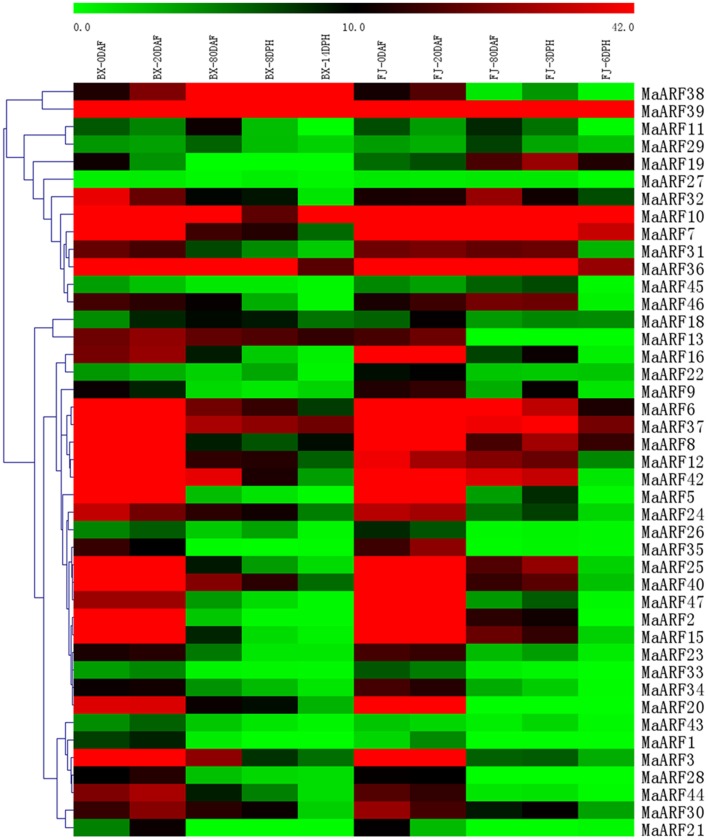
**Expression analysis of MaARF genes in different stages of fruit development and ripening in two banana varieties**. Fruits of 0, 20, and 80 DAF in BX and FJ varieties were sampled to assess the expression patterns of MaARF genes during fruit development. Fruits at 8 and 14 DPH in BX and at 3 and 6 DPH in FJ were sampled to examine the expression patterns of MaARF genes in the post-harvest ripening process. The heat map was created based on the transcriptomic data of ARFs from two independent experiments. The scale represents the signal intensity values.

Auxin has been shown to play a crucial role in controlling fruit development (Serrani et al., 2008; de Jong et al., 2009). Some studies have shown a function for *ARF* genes in fruit development and ripening (de Jong et al., 2009; Kumar et al., 2011; Sagar et al., 2013; Wan et al., 2014; Zouine et al., 2014). Thus, there is a need to investigate the transcriptional response of *MaARF* genes during fruit development and ripening for the banana. At 0, 20, and 80 DAF of fruits of the BX and FJ varieties, at 8 and 14 DPH of fruits in BX, and at 3 and 6 DPH of fruits in FJ, tissues were sampled to measure the expression patterns of the *MaARF* genes. Transcriptional analyses revealed the expression levels of 43 *MaARF* genes at different stages of fruit development and ripening (Figure 5, Table S4).

For BX, the expression of all 43 *MaARF* genes could be detected at 0, 20, and 80 DAF and at 8 DPH, whereas 41 *MaARF* genes (not *MaARF-1* or *-11*) showed transcript accumulation at 14 DPH. There were 18 (41.9%), 17 (39.5%), 6 (*MaARF-10, -36, -37, -38, -39*, and *-42*; 14.0%), 3 (*MaARF-36, -38*, and *-39*; 7.0%), and 3 (*MaARF-10, -38*, and *-39*; 7.0%) *MaARF* genes that showed high expression levels (value > 30) at 0, 20, and 80 DAF, and at 8 DPH and 14 DPH, respectively. Notably, *MaARF39* exhibited high expression levels (value > 30) at all stages of fruit development and ripening.

For FJ, 43, 43, 40 (not *MaARF-28, -13*, or *-20*), 40 (not *MaARF-28, -13*, or *-20*), and 38 (not *MaARF-1, -28, -33, -13*, or *-20*) *MaARF* genes expressed at 0, 20, and 80 DAF, and at 3 and 6 DPH, respectively. Furthermore, 19 (44.2%), 19 (44.2%), 7 (*MaARF-6, -7, -10, -36, -37, -39*, and *-42*; 16.3%), 8 (*MaARF-6, -7, -8, -10, -36, -37, -39*, and *-42*; 18.6%), and 3 (*MaARF-7, -10*, and *-39*; 7.0%) *MaARF* genes showed high transcript accumulation (value > 30) at 0, 20, and 80 DAF, and at 3 and 6 DPH, respectively. *MaARF-7, -10*, and *-39* exhibited high expression levels (value > 30) at all stages of fruit development and ripening.

In a comparison of the expression profiles of *MaARFs* at different stages of fruit development and ripening in BX and FJ, 41 *MaARF* genes (95.3%) showed expression at all stages tested in BX, whereas 38 *MaARF* genes (88.4%) showed expression at all stages examined in FJ. Generally, similar expression patterns could be observed at 0 and 20 DAF in BX and FJ, indicating that the *MaARF* genes played similar roles in these two fruit developmental stages in BX and FJ. However, 10 genes (*MaARF-6, -7, -8, -15, -19, -25, -31, -32, -37*, and *-46*) had higher expression levels at subsequent stages in FJ compared to those in BX. These findings implied a significant transcriptional response during the post-harvest ripening of FJ. Additionally, *MaARF39* exhibited high expression levels (value > 30) at all stages in BX and FJ.

### Expression profiles of *MaARF* genes in response to cold, salt, and osmotic stresses in two banana varieties

**Figure 6:**
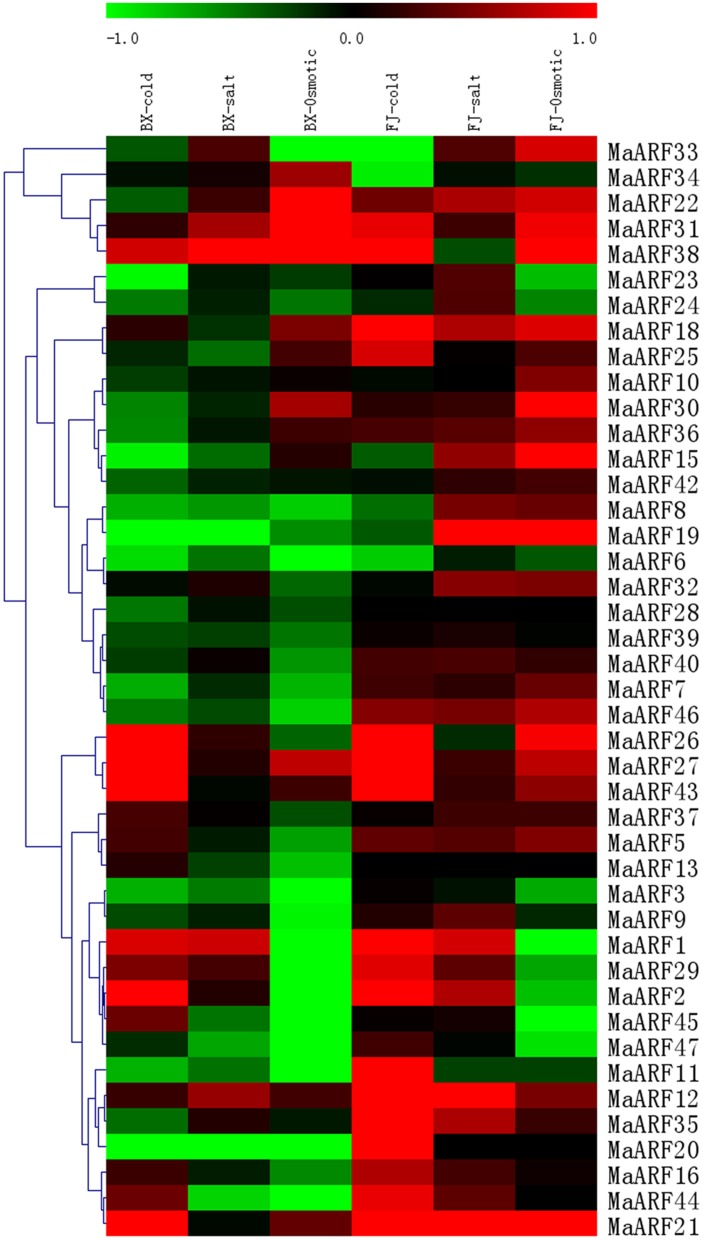
**Expression analysis of MaARF genes in response to cold, salt, and osmotic treatments in two banana varieties**. For osmotic and salt treatments, banana seedlings at the five-leaf stage grown in soil were irrigated with 200 mM mannitol or 300 mM NaCl for 7 days, respectively. For cold treatment, banana seedlings were cultured in a growth chamber with a temperature that was maintained at 4°C for 22 h. Log_2_ based value was used to create the heat map based on the transcriptomic data of ARFs from two independent experiments. The scale represents the relative signal intensity values.

Extensive research has shown that various environmental signals are integrated into changes in auxin homeostasis, redistribution, and signaling (Navarro et al., 2006; Park et al., 2007; Shibasaki et al., 2009; Chen et al., 2010). To better understand the relationship between auxin signal transduction and abiotic stresses, the expression of MaARF genes in leaves of BX and FJ was examined when banana plants were subjected to cold, salt, or osmotic treatments. Transcriptomic analyses showed the expression of 43 and 41 (not *MaARF-13* or *-28*) *MaARF* genes in BX and FJ varieties, respectively (Figure 6, Table S5).

For BX, 17 (39.5%), 15 (34.9%), and 14 (32.6%) *MaARF* genes were upregulated by cold, salt, or osmotic stress, respectively. By contrast, 26 (60.5%), 28 (65.1%), and 29 (67.4%) *MaARF* genes were downregulated by cold, salt, and osmotic stress, respectively. There were 5 (*MaARF-2, -26, -27, -43*, and *-21*), 1 (*MaARF-38*), and 3 (*MaARF-22, -31*, and *-38*) *MaARF* genes that were significantly induced (value > 1) after cold, salt, or osmotic stress, respectively, as well as 4 *MaARF* genes (*MaARF-12, -27, -31*, and *-38*) that showed upregulation in response to each of the stress treatments.

For FJ, 31 (75.6%), 33 (80.5%), and 26 (63.4%) *MaARF* genes were upregulated by cold, salt, and osmotic stress, respectively. By contrast, 10 (24.4%), 7 (17.1%), and 14 (34.1%) *MaARF* genes were downregulated by cold, salt, and osmotic stress, respectively. Additionally, 12, 3 (*MaARF-12, -19*, and *-21*), and 5 (*MaARF-15, -19, -30, -38*, and *-21*) *MaARF* genes showed significant induction (value > 1) after cold, salt, and osmotic stress, respectively, and 17 *MaARF* genes showed upregulation in response to each of the stress treatments.

Based on these findings, we can state that the numbers of upregulated and significantly upregulated MaARF genes by abiotic stresses were greater in FJ than in BX. Moreover, 3 MaARF genes (MaARF-12, -27, and -31) showed upregulation in response to cold, salt, and osmotic stress in both BX and FJ. However, MaARF-6 showed downregulation in response to cold, salt, and osmotic stress in both BX and FJ.

### ARF family interaction networks and their co-expression during early fruit development

**Figure 7:**
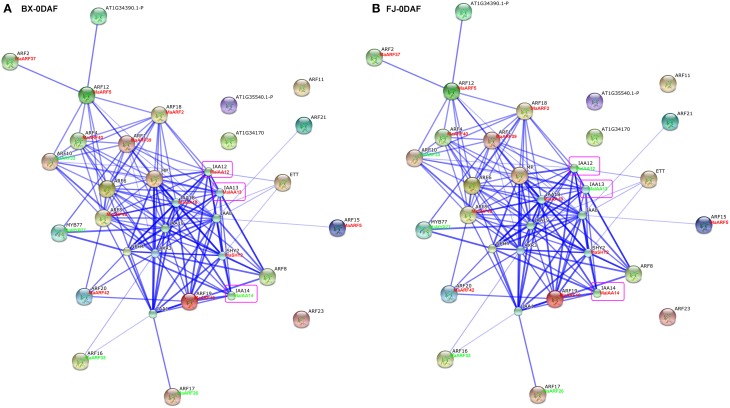
**Interaction network and co-expression analyses of ARF genes at 0 DAF stage of fruits in BX (A) and FJ (B) varieties of banana and related genes in Arabidopsis**. Line thickness relates to combined score. The homologous genes of banana are in parentheses. The genes marked with red font show high expression levels (value > 10). The genes marked with green font show low expression levels (value < 10). The genes boxed by purple indicate their differential expression patterns between BX and FJ.

**Figure 8:**
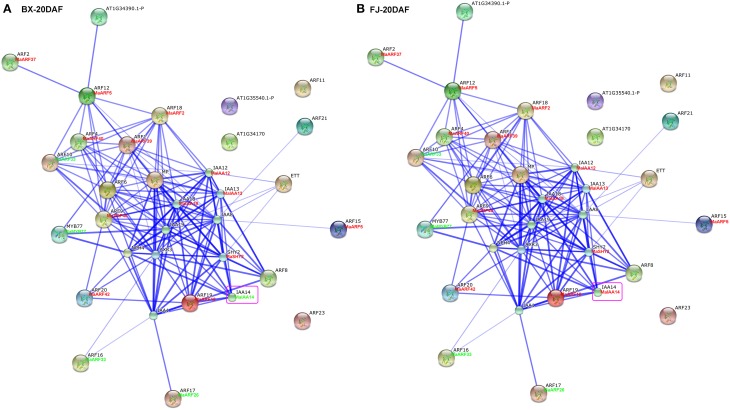
**Interaction network and co-expression analyses of ARF genes at 20 DAF stage of fruits in BX (A) and FJ (B) varieties of banana and related genes in Arabidopsis**. Line thickness relates to combined score. The homologous genes of banana are in parentheses. The genes marked with red font show high expression levels (value >10). The genes marked with green font show low expression levels (value < 10). The genes boxed by purple indicate their differential expression patterns between BX and FJ.

Studies of gene family interaction networks are very useful for investigating gene functions (Tohge and Fernie, 2010). Because most ARFs contain a CTD domain that is involved in protein–protein interactions, the identification of potential interaction networks of banana ARFs will aid future studies of their biological function based on experimentally validated interactions. STRING software was used to construct ARF-mediated networks, and we found that 10 interactive proteins (with high confidence; score > 0.9) were involved in ARF family networks in *Arabidopsis* (Figure S1). These ARF partners include 9 Aux/IAAs and 1 MYB transcription factors. Furthermore, homologs of these interaction proteins in banana were identified by reciprocal BlastP analyses and the expression profiles of these genes during early fruit development of BX and FJ were examined based on RNA-seq data sets. At 0 DAF of fruits in BX, we found 24 protein–protein interactions showing high expression levels (value > 10), such as, ARF2:MaARF37-ARF12:MaARF5, ARF12:MaARF5-ARF18:MaARF2, ARF12: MaARF5-ARF4:MaARF40, ARF12:MaARF5-ARF1:MaARF39, ARF18:MaARF2-ARF4: MaARF40, ARF18:MaARF2-ARF1:MaARF39, ARF18:MaARF2-IAA12:MaIAA12, ARF18:MaARF2-IAA13:MaIAA13, and ARF18:MaARF2-IAA18:MaIAA18 (Figure 7A, Tables S6, S7). At 0 DAF of fruits in FJ, the co-expression patterns was similar to that in BX, in which only three genes MaIAA12, MaIAA13, and MaIAA14 showed differential expression patterns relative to that in BX (Figure 7B, Table S6). At 20 DAF of fruits in BX, the identified co-expression patterns were the same as 0 DAF of fruits in BX (Figure 8A, Table S6). Compared with co-expression patterns of 20 DAF of fruits in BX, only MaIAA14 showed different expression profile at 20 DAF of fruits in FJ (Figure 8B, Table S6). Collectively, these results indicated that ARF family interaction networks had similar co-expression patterns with most genes showing high expression levels during early fruit development stages in BX and FJ, implying a crucial role for these genes in fruit development. The interaction networks and co-expression patterns identified in this study will contribute to further investigating the exact roles that ARF genes play in the fruit development in banana.

## Discussion

The banana, a typical climacteric fruit, is one of the most popular fresh fruits enjoyed worldwide. The mechanisms involved in banana fruit development and ripening, and responses to abiotic stresses remain poorly characterized. The auxin signal pathway has been shown to play important roles in various aspects of plant growth and development, and environmental adaptation, among which the ARF gene family is important transcription factors for the auxin signaling pathway (Davies, 1995; Park et al., 2007; Zhang et al., 2009; Zahir et al., 2010; Du et al., 2012; Lee et al., 2012). ARFs were also reported to be involved in the regulation of plant growth and development, and responses to abiotic stresses in various species. However, the identity and function of banana ARFs have remained unknown. Herein, we identified 47 ARF family members in banana genome and showed possible roles for MaARFs in banana development, ripening, and responses to abiotic stresses, which will establish a solid foundation for further studies of the function of *ARF* genes and the ARF-mediated auxin signaling pathway in banana.

### Identification and molecular characterization of the banana *ARF* gene family

The genome sequence of DH-Pahang (*Musa acuminate*, A-genome, 2*n* = 22) was used as a reference to identify all members of the ARF gene family and 47 ARF genes were identified in our genome-wide analysis. These findings suggested that the ARF gene family in banana had expanded compared to that in *Arabidopsis* (23) and rice (25) (Okushima et al., 2005; Wang et al., 2007). An evolutionary analysis showed that the banana ARFs could be divided into four subgroups, which is in accordance with previous phylogenetic classifications of ARFs in rice (Wang et al., 2007), soybean (Ha et al., 2013), *Eucalyptus grandis* (Yu et al., 2014), Tomato (Zouine et al., 2014), *Arabidopsis* (Okushima et al., 2005), and *Brassica rapa* (Mun et al., 2012). Generally, ARFs from the banana were more closely related to those from rice than those from *Arabidopsis*, which is consistent with the currently accepted view of plant evolution (Okushima et al., 2005; Wang et al., 2007) (Figure 1). Our gene structure analysis indicated that the number of exons of *MaARFs* was greater in clusters I–III than in cluster IV (Figure 2). This phenomenon was also observed in other species, such as *Arabidopsis*, rice, and *Brassica rapa* (Okushima et al., 2005; Wang et al., 2007; Mun et al., 2012). Thus, the exon numbers in each group among all four species supports their evolutionary relationship and the current group classification scheme. Moreover, compared with the exon numbers of *ARF* genes in other species, such as *Arabidopsis* (2–15) (Okushima et al., 2005), rice (3–16) (Wang et al., 2007), and *Brassica rapa* (2–16) (Mun et al., 2012), more exons were observed for banana *ARF* genes (Figure 2). The large structural variation of banana *ARF* genes implied that the banana genome changed significantly during its evolutionary history. A conserved motifs analysis revealed that all MaARFs had a typical DBD domain required for efficient binding to AuxRE (Figure 3, Table S2) (Hagen and Guilfoyle, 2002; Ha et al., 2013). Additionally, most MaARFs contained a CTD domain, which can mediate the dimerization of ARFs or ARF and Aux/IAA protein (Tiwari et al., 2003; Guilfoyle and Hagen, 2007). The percentage of CTD-truncated MaARFs (25.5%) was similar to that of ARFs in rice (24%) *Brassica rapa* (22.6%), and tomato (28.6%) (Shen et al., 2010; Wu et al., 2011; Mun et al., 2012). Together, the identification and classification of the MaARF gene family was supported by evolutionary, gene structure, and conserved motif analyses.

### The transcriptional response of *ARF* genes during banana fruit development and post-harvest ripening

One of the vital traits of the banana is fruit quality, which is determined by fruit development prior to harvest and the post-harvest ripening process. Thus, scientific interest in banana has been mostly focused on development- and ripening-related processes that are important for enhancing the commercial export potential of this fruit (Bapat et al., 2010). However, it remains unclear whether *ARF* genes are involved in banana development and ripening. In the present study, the expression patterns of *MaARF* genes in fruit development and ripening processes were examined, and suggested that most *ARF* genes showed changes at the transcriptional level. Moreover, many *MaARF* genes showed robust expression levels (value > 30) during these developmental and ripening stages (Figure 5, Table S4). Our results suggested that *MaARF* genes might be the involved in banana fruit development and ripening. Similar expression changes amongst *ARF* genes were also observed in grape and tomato. In grape, the expression of 6 of 9 *ARF* genes changed significantly during berry development (Wan et al., 2014). In tomato, most *SlARFs* have been reported to undergo a strong change in expression associated with the flower-to-fruit transition and subsequent ripening process (Zouine et al., 2014). It is concluded that *ARF* genes may extensively participate in regulating the fruit development and ripening processes.

Interestingly, the number of *MaARF* genes with high expression levels (value > 30) at the 0 and 20 DAF stages is greater than that at the remaining stages, suggesting crucial roles for the *ARF* genes in early banana fruit development (Figure 5, Table S4). Previously, biochemical and genetic studies revealed the importance of *ARFs* during early fruit development (Goetz et al., 2006; de Jong et al., 2009, 2011). For example, tomato *SlARF7* transcripts showed high levels in unpollinated mature ovaries and increased during flower development. Transgenic plants with reduced *SlARF7* mRNA levels yielded seedless fruits (de Jong et al., 2009). Accordingly, a mutation in *ARF8* resulted in the uncoupling of fruit development from pollination and fertilization and gives rise to seedless fruit in *Arabidopsis* (Goetz et al., 2006). Together, these findings demonstrate the critical roles of *ARFs* during early fruit development. Auxin has been shown to play a crucial role in controlling early fruit development (Serrani et al., 2008; de Jong et al., 2009), representing an important step in the development of higher plants. Given the predominant role of auxin signaling in fruit development (Serrani et al., 2008; de Jong et al., 2009), the robust expression of these *MaARF* genes implies their potential involvement in mediating auxin responses during early banana fruit development.

The banana, a typical climacteric fruit, undergoes a post-harvest ripening process characterized by a green-storage phase. In this process, many physiological and biochemical changes occur in banana fruit, including chlorophyll breakdown, flavochrome accumulation, and degradation of cell wall components, which result in fruit softening and the degradation of stored starch into soluble sugar (Thomas and Janave, 1992; Hill and Rees, 1995a,b; Jacob-Wilk et al., 1999; Lohani et al., 2004; Hu et al., 2014). As a result, post-harvest ripening plays an important role in improving fruit quality and extending fruit shelf life. Although genome-wide expression and functional analyses also demonstrated roles for *ARFs* in the fruit ripening process prior to harvest (Sagar et al., 2013; Zouine et al., 2014), it remains unknown whether *ARFs* are involved in the regulation of the post-harvest ripening process. Herein, we detected transcriptional changes of *MaARF* genes during the post-harvest banana ripening process, implying the involvement of *ARF* genes in banana ripening. Notably, 10 genes (*MaARF-6, -7, -8, -15, -19, -25, -31, -32, -37*, and *-46*) had higher expression levels at post-harvest banana ripening stages in FJ than in BX, indicating a significant response at the transcriptional levels in the post-harvest ripening of FJ (Figure 5, Table S4). Moreover, FJ ripened faster than BX during post-harvest ripening. BX required 8 and 14 DPH to reach MG and FY degrees, respectively, whereas FJ required 3 and 6 DPH to reach MG and FY degrees, respectively. These findings suggested that *ARF* genes might be involved in promoting post-harvest banana ripening, and supports a potential link between auxin signaling and post-harvest banana ripening.

### The transcriptional response of *ARF* genes in banana abiotic stresses response

The banana is considered to be extremely sensitive to water stress, such as those driven by drought, salt, or cold, so it requires an abundant water supply because of its permanent green canopy, shallow roots, and rapid growth rate (van Asten et al., 2011). However, few studies have been carried out on banana responses to abiotic stresses, along with related signaling behaviors (Sreedharan et al., 2013). Genome-wide expression analyses have suggested that the expression of numerous *ARF* genes change when plants respond to abiotic stresses in various species (Jain and Khurana, 2009; Wang et al., 2010; Ha et al., 2013; Sekhwal et al., 2015). Recently, tasiRNA-ARF was reported to be involved in maintaining normal flower morphogenesis under stress conditions by fine-tuning expression changes in floral development- and auxin response-related genes in *Arabidopsis* (Matsui et al., 2014). Therefore, accumulating evidence indicates that auxin plays a role in plant responses to abiotic stresses through complex metabolic and signaling networks. In this study, we found that many *MaARF* genes can respond to abiotic stresses, including osmotic, salt, and cold stresses, at the transcriptional level in both varieties, indicating essential roles of these genes in responsive to abiotic stresses in banana. Furthermore, we also observed that the number of both upregulated (value > 0) and significantly upregulated (value > 1) *MaARF* genes by abiotic stresses were greater in FJ than in BX (Figure 6, Table S5). Cultivated bananas originate either from intraspecific hybridizations between wild diploid subspecies of *Musa acuminata* (A-genome) or from interspecific crosses between *M. acuminata* and the wild diploid *M. balbisiana* (B-genome). The B-genome (*M. balbisiana*) has been associated with improved vigor and tolerance to both biotic and abiotic stresses, and so it is therefore a target for banana breeding programs (Davey et al., 2013). Most banana varieties containing a B-genome display strong resistance to water stress (Bananuka et al., 1999; Huang et al., 1999). Collectively, it is concluded that *ARF* genes might play a role in the regulation of banana resistance to abiotic stresses in banana. These findings establish a solid foundation for further studies of the auxin-mediated abiotic stresses response in banana.

## Conclusion

In the present study, we identified 47 banana *ARF* genes and established the classification and evolutionary relationship of these genes using phylogenetic, gene structure, and conserved protein motif analyses. Expression analyses revealed the involvement of *MaARF* genes in banana growth, fruit development, post-harvest ripening, and responses to abiotic stresses. Additonally, a comparison of the differential expression profiles of *MaARFs* in BX and FJ suggested that some *MaARF* members might contribute differentially to fruit development, post-harvest ripening, and banana abiotic stress responses. Furthermore, interaction networks and co-expression assays indicated the strong transcriptional response of banana ARFs and ARF-mediated networks in early fruit development for different varieties, implying a crucial role for these genes in fruit development. These data will provide a solid foundation for future studies of the functional characterization of *ARF* genes and ARF-mediated signal transduction pathways, thereby advancing our understanding of the molecular basis of genetic enhancements to the banana. Because auxin-mediated signal transduction pathway is complex, further systematic studies on the target genes of ARF in auxin signaling pathway, such as SAUR, GH3, and AUX/IAA are required to increase our understanding of the development, ripening and environmental adaptation of banana.

## Availability of supporting data

The sequences of banana *ARF* genes identified in this study could be available in banana genome database (http://banana-genome.cirad.fr/) (The accession numbers were listed in Table S1).

## Author contributions

BX and ZJ conceived the study. WH, JZ, XH, YY, YW, JL, and ML performed the experiments and carried out the analysis. WH, JZ, and XH designed the experiments and wrote the manuscript. All authors read and approved the final manuscript.

### Conflict of interest statement

The authors declare that the research was conducted in the absence of any commercial or financial relationships that could be construed as a potential conflict of interest.

## Supplementary material

Figure S1 **ARF-mediated interaction networks in *Arabidopsis***.

Table S1 **Characteristics of ARFs in banana**.

Table S2 **Conserved amino acid motifs and annotation of MaARFs**.

Table S3 **The expression data of the banana ARF genes in different tissues of BX and FJ varieties**.

Table S4 **The expression data of the banana ARF genes in different stages of fruit development and ripening in BX and FJ varieties**.

Table S5 **The expression data (log_2_-based values) of the banana ARF genes after various abiotic stress treatment in BX and FJ**.

Table S6 **Expression data of the genes involved in ARF-mediate interaction networks during fruit development in BX and FJ**.

Table S7 **Protein–protein interactions with high expression levels (value > 10) at 0 DAF of fruits in BX**.
