# Supplementary figures and images for: The auxin response factor gene family in banana: genome-wide identification and expression analyses during development, ripening, and abiotic stress
Source: Front Plant Sci. 2015 Sep 15;6:742. doi: 10.3389/fpls.2015.00742 (PMC4569978; doi:10.3389/fpls.2015.00742)

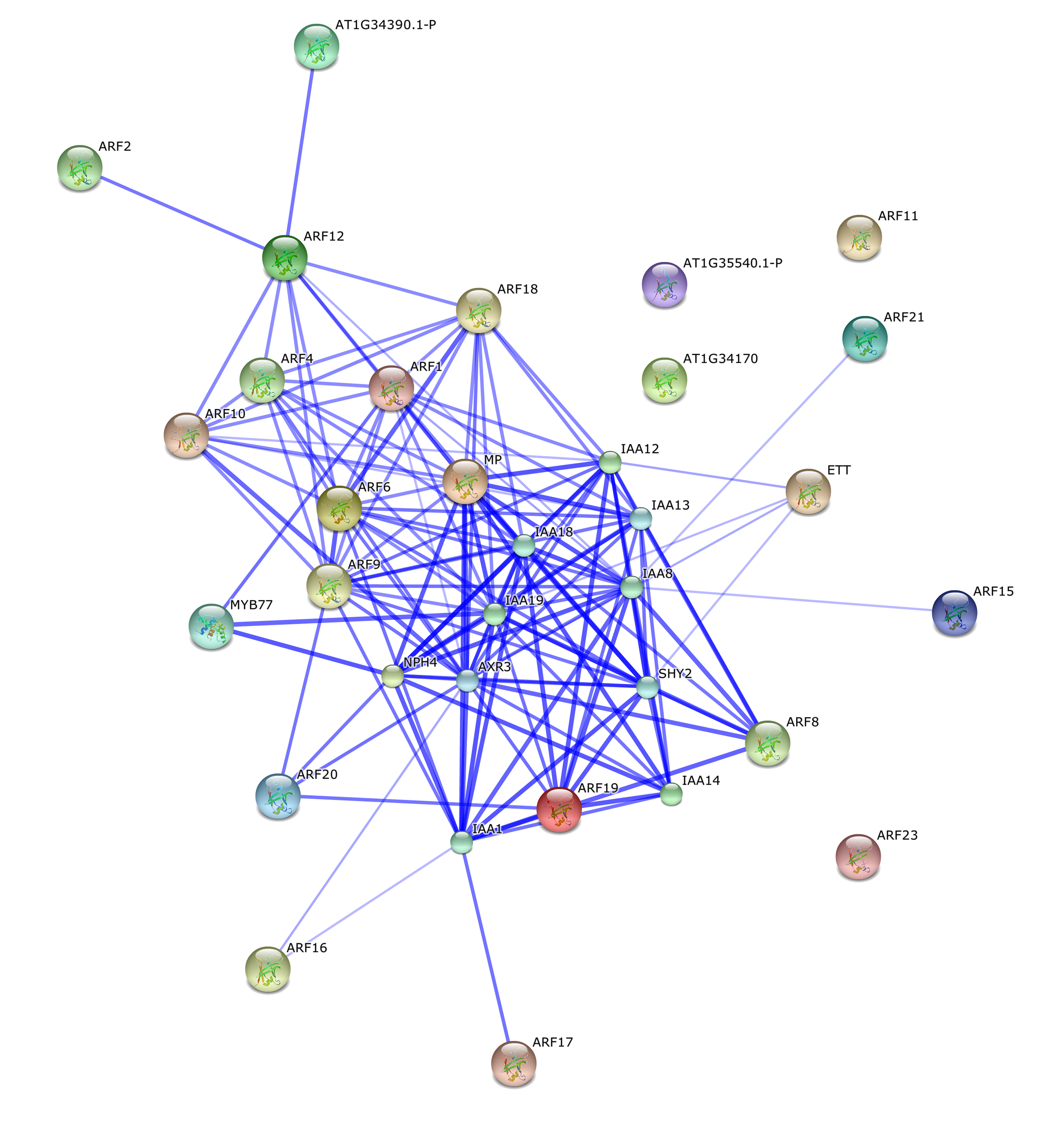

Supplement: Figure S1 — ARF-mediated interaction networks in Arabidopsis. [file Image1.TIF]
